# Evaluation of a Low-Threshold/High-Tolerance Methadone Maintenance Treatment Clinic in Saint John, New Brunswick, Canada: One Year Retention Rate and Illicit Drug Use

**DOI:** 10.1155/2013/753409

**Published:** 2012-10-23

**Authors:** Timothy K. S. Christie, Alli Murugesan, Dana Manzer, Michael V. O'Shaughnessey, Duncan Webster

**Affiliations:** ^1^Ethics Services, Horizon Health Network, Miramichi, NB, Canada, E1V 1Y3; ^2^Department of Bioethics, Dalhousie University, Halifax, NS, Canada, B3K 6R8; ^3^Dr. Reiman's Cancer Research Laboratory, University of New Brunswick, Saint John, NB, Canada, E3B 5A3; ^4^Uptown Clinic, St. Joseph's Hospital, Saint John, NB, Canada, E2L 3L6; ^5^British Columbia Centre for Excellence in HIV/AIDS, Vancouver, BC, Canada, V6Z 1Y6; ^6^Department of Medicine, Saint John Regional Hospital, Saint John, NB, Canada, E2L 3L6

## Abstract

*Objective*. To report the one-year retention rate and the prevalence of illicit opioid use and cocaine use in the Low-Threshold/High-Tolerance (LTHT) methadone maintenance treatment (MMT) clinic located in Saint John, New Brunswick, Canada. *Methods*. A description of the LTHT MMT clinic is provided. The one-year retention rate was determined by collecting data on patients who enrolled in the LTHT MMT clinic between August 04, 2009 and August 04, 2010. The prevalence of illicit drug use was determined using a randomly selected retrospective cohort of 84 participants. For each participant the results of six consecutive urine tests for the most recent three months were compared to the results of the first six consecutive urine tests after program entry. *Results*. The one-year retention rate was 95%, 67% of the cohort achieved abstinence from illicit opioids and an additional 13% abstained from cocaine use. *Conclusion*. The novel feature of the LTHT MMT clinic is that patients are not denied methadone because of lack of ancillary services. Traditional comprehensive MMT programs invest the majority of financial resources in ancillary services that support the biopsychosocial model, whereas the LTHT approach utilizes a medical model and directs resources at medical management.

## 1. Introduction

 Most Canadian methadone maintenance treatment (MMT) programs have limited treatment capacity, hundreds of people on wait-lists and wait-times that exceed 6–12 months [1]. The result is that many people, who are motivated to seek treatment for their addiction, remain untreated. In Canada, the provision of MMT is regulated by Health Canada's guidelines, with each province and/or territory having the option of developing their own [2]. A review of the Federal Guidelines and those of seven provinces demonstrate that each adheres to the biopsychosocial model of MMT and require, among other things, the provision of psychosocial counseling, random urine testing, and mechanisms for the involuntary discharge of patients [3–9].

 This paper will report the evaluation results of the low-threshold/high-tolerance (LTHT) MMT clinic in Saint John, New Brunswick, Canada. The LTHT approach is novel because it utilizes a medical model rather than the traditional biopsychosocial model that predominates in Canada. This model incorporates recommendations of the Government of Canada “best practices” document with the foundational premise that the provision of MMT should not be contingent on the availability of resources for psychosocial treatment [10, 11].

 The “low-threshold” aspect refers to the removal of barriers that limit or delay access to MMT. The referral process is open so clients can be referred from any source, including self-referral. Intake assessments are minimized; patients undergo a basic biopsychosocial evaluation and are admitted sequentially from the wait-list (although patients may be triaged in serious situations such as pregnancy). The “high-tolerance” aspect focuses on strategies designed to retain patients in treatment, for example, there is no mandated group or individual counseling, urine tests are scheduled not random, and the results are not used punitively as there is a “no involuntary discharge policy” relating to continued illicit drug use.

## 2. Objective

The objective is to report the one-year retention rate and the prevalence of illicit opioid use and cocaine use in the LTHT MMT clinic located in Saint John, New Brunswick, Canada. 

## 3. Methods

### 3.1. One-Year Retention Rate

The one-year retention rate was determined by collecting data on each patient who received MMT from the LTHT clinic between August 04, 2009 and August 04, 2010. The total number of patients enrolled in the clinic during this time period was compared to the number of patients still in the program after 12 months. If a patient was no longer receiving methadone from the clinic, the reason(s) for his or her separation were determined. This was an intent-to-treat analysis, for example, regardless of whether the patient was on a stable dose of methadone or not, all patients who entered treatment during this time frame were included in the denominator, and only those still receiving MMT after 12 months were included in the numerator.

### 3.2. Illicit Opioid and Cocaine Use

The methods used to measure illicit opioid and cocaine use included (1) the development of inclusion and exclusion criteria, (2) the development of outcome measures, and (3) statistical analysis. 

*Inclusion and Exclusion Criteria*. To measure the impact of the LTHT MMT clinic on illicit opioid and cocaine use, it was necessary to develop inclusion criteria that could control for variables that might impact on a participant's illicit drug use. For example, if a person was transferred to the LTHT MMT clinic from another MMT program, he or she might be abstinent from illicit drugs at program entry. Thus, the absence of illicit drug use could be explained independly of receiving treatment via the LTHT MMT clinic. Likewise, during the methadone induction phase (the period of time when a patient is being titrated to an adequate methadone dose), the patient will not yet receive the full benefit of the methadone medication; continued illicit drug use is to be expected during this time period. 

 To control for this variability, the following inclusion and exclusion criteria were developed: (1) a participant could not be on a stable dose of methadone at program entry, and (2) participants must be on a stable dose of methadone after receiving treatment from the LTHT MMT clinic. A participant was considered to be on a stable dose of methadone at program entry (and ineligible for the study) if he or she had been transferred from another methadone program and had not received a single methadone dose adjustment greater than 10 mg within the first three months of entering the LTHT MMT clinic. Second, a patient was considered to be on a stable dose of methadone if he or she had been in treatment at the LTHT MMT clinic and had not received a methadone dose adjustment greater than 10 mg in the most recent three months. 

*Outcome Measurement.* For each research participant, the results of six consecutive urine tests for the most recent three months were compared to the results of that participant's first six consecutive urine tests for the first three months after program entry; there was no overlap between these two time intervals. It was determined how many times each participant tested positive for illicit opioids or cocaine: one out of six times, two out of six times, three out of six times, four out of six times, five out of six times, or six out of six times. These results were compared to the same participants' first six urine test results at program entry. 

*Statistical Analysis.* Paired *t*-tests were conducted to determine whether the mean number of positive tests was different between these two time intervals; an effect size was calculated using Cohen's D, and the alpha level was set at *P* < 0.05.

## 4. Results

### 4.1. One-Year Retention Rate

Between August 04, 2009 and August 04, 2010, 179 patients were enrolled in the LTHT MMT clinic. Uptake was immediate and dramatic: 122 patients entered treatment during the first three months of operation, and 153 were enrolled within five months. The average methadone dose was 70 mg; 66% were men, and the average age was 35 years. Of the 179 patients enrolled in the LTHT MMT clinic during the study period, 95% (*n* = 170) were still receiving methadone after one year. Of the nine people separated from the clinic at the time of analysis, three had voluntarily withdrawn their participation, three were incarcerated, two were transferred to primary care providers for continued MMT, and one person went into the witness protection program. Of the three people who voluntarily withdrew, two were females, their ages were 30, 45, and 47 years, and they were in treatment for 4, 7, and 10 months. The three patients incarcerated at the time of analysis were maintained on methadone while in jail and were to be readmitted to the LTHT MMT clinic upon their release, and the two patients who were transferred to other MMT providers continued to receive prescribed methadone. No information is available on the person who went into the witness protection program (for obvious reasons).

### 4.2. Randomly Selected Retrospective Cohort

Table 1 shows that out of the 179 patients in the LTHT MMT clinic, nine patients were separated from the clinic, eight were stable transfers from another MMT clinic, 28 were in the induction phase of treatment, 134 met the inclusion criteria (i.e., they were unstable at program entry and subsequently reached a stable dose of methadone), and 84 were randomly selected to be in the study. The random selection process started with a complete list of the 134 stable patients, the list was shuffled so that the order was random, then a random number generator produced a random list, and the corresponding patient was selected for inclusion until a sample of 84 people was generated. 

All participants gave informed consent, and no one refused the invitation to participate. The average length of time in treatment was 16.2 months (range 9–18 months), the average methadone dose was 72.75 mg, 62% were men, and the average age was 35 years. These participants constitute 47% of all patients ever enrolled in the LTHT MMT clinic during the study period, and they are representative of the entire LTHT MMT clinic patient population in terms of age, gender, length of time in treatment, and methadone dose.

#### 4.2.1. Opioid Positive Urine Tests

 At program entry, the prevalence of illicit opioid use was 100%. Following entry into the program, initial urine screening showed that 23 participants tested positive for illicit opioids 1/6 times, 21 tested positive 2/6 times, 12 tested positive 3/6 times, 11 tested positive 4/6 times, five tested positive 5/6 times, and 12 tested positive 6/6 times. After stabilizing on methadone, the prevalence of illicit opioid use decreased to 33%: 56 research participants tested positive for illicit opioids 0/6 times, 15 tested positive 1/6 times, three tested positive 2/6 times, two tested positive 3/6 times, two tested positive 4/6 times, five tested positive 5/6 times, and one person tested positive 6/6 times. These participants were more likely to test positive for illicit opioids before stabilizing on methadone (mean = 2.88, SE = 0.19) than after stabilizing on methadone (mean = 0.79, SE = 0.15), *t*(83) = −11.51, *P* < .001, Cohen's D = −1.29. The decrease in positive tests was statistically significant (*P* < .001), and the effect size of −1.29 is considered large.

 A comparison between the 56 research participants who consistently tested negative for illicit opioids with the 28 participants who tested positive at least once reveals no difference in methadone dose. The average methadone dose was 72.5 mg for those consistently testing negative and 73.21 mg for those testing positive at least once. A Mann-Whitney *U* Independent Samples Test determined that there is no statistically significant difference between these means (*P* = .924).

#### 4.2.2. Cocaine Positive Urine Tests

At program entry, the prevalence of cocaine use was 55%. Following entry into the program, initial urine screening showed that 38 participants tested positive for cocaine 0/6 times, eight tested positive 1/6 times, six tested positive 2/6 times, four tested positive 3/6 times, six tested positive 4/6 times, nine tested positive 5/6 times, and 13 tested positive 6/6 times. After stabilizing on methadone the prevalence of positive cocaine tests decreased to 43%: 48 research participants tested positive for cocaine 0/6 times, eight tested positive 1/6 times, five tested positive 2/6 times, three tested positive 3/6 times, four tested positive 4/6 times, four tested positive 5/6 times, and 12 people tested positive 6/6 times. Although methadone has no biological effect on cocaine use, participants were more likely to test positive for cocaine before stabilizing on methadone (mean = 2.16, SE = 0.263) than after stabilizing on methadone (mean = 1.63, SE = 0.252), *t*(83) = −2.56, *P* = .012, Cohen's D = −0.23. The decrease in positive cocaine tests was statistically significant (*P* = .012), and the effect size of −0.23 is considered small.

## 5. Discussion

### 5.1. One-Year Retention Rate

Since there are no published data regarding the traditional biopsychosocial model in New Brunswick it is not possible to formally compare the two local models. However, a comparison may be made to the rates in British Columbia where they report a one-year retention rate between 40.5% and 52% [12–14, 16]. More recently the North American Opiate Medication Initiative (NAOMI) published an 87.8% one-year retention rate for the heroin prescription arm and a 54.1% one-year retention rate for the methadone arm [15]. The methadone arm in NAOMI had a “no involuntary discharge policy,” which is a unique feature of the LTHT clinic making them somewhat comparable. However, an important qualification is that 92% of the illicit opioid use in Saint John, New Brunswick, involves the illegal use of prescription opioids for nonmedical purposes [16]. British Columbia has higher rates of heroin use, and NAOMI focussed exclusively on heroin users. Thus, there might be important differences among these patient populations that make direct comparisons inappropriate. Nevertheless, addiction to prescription opioids has been identified as an emerging epidemic in North America, and methadone is the appropriate medication for this condition [14, 17].

### 5.2. Illicit Drug Use

The 67% reduction in illicit opioid use is to be expected in a MMT program. In fact, the literature on this point is conclusive [18–21]. Interestingly, however, there is no difference in the average methadone dose between those patients who continued to use illicit opioids and those who abstained completely. Since there is no arbitrary limit to the methadone dose, continued opioid use among these patients is not likely to be attributed to inadequate dosing. Finally, there is evidence that retention in treatment and reduction in illicit opioid use are positively correlated with decreases in cocaine use, which is what has been observed in this cohort [22, 23].

 In this study, the results of urine tests were used as a proxy measure for illicit opioid and cocaine use, with the tests being scheduled as opposed to random. Although there were no negative consequences associated with testing positive for illicit drugs, consistent with the “no involuntary discharge policy,” it is possible that patients abstained from illicit drug use prior to submitting a urine specimen. For example, 95% of cocaine metabolite is excreted within 48 hours and 100% by 72 hours irrespective of the dose consumed. Thus, patients could have used cocaine prior to 72 hours before submitting to urine testing. Likewise, the opiate assay was designed to measure codeine, morphine, and hydromorphone at concentrations above 300 ng/mL, so lower concentrations may not have been detected by this test in some instances. Finally, since the current study did not have a control group, additional research is required in order to determine how the LTHT approach compares to other models of providing MMT. Ultimately, a well-designed controlled prospective clinical trial that formally compares the LTHT approach to differing MMT treatment modalities is required. 

## 6. Conclusion

 The one-year retention rate for the LTHT MMT clinic is 95%; 67% of participants achieved abstinence from illicit opioids, and an additional 13% abstained from cocaine use after stabilizing on methadone. The novel feature of the LTHT MMT clinic is that patients are not denied methadone because of lack of ancillary services. Traditional comprehensive MMT programs invest the majority of financial resources in ancillary services that support the biopsychosocial model, whereas the LTHT approach utilizes a medical model and directs resources at medical management. It is important to note, however, that patients at the LTHT clinic can receive ancillary services from other providers at the Uptown Clinic. For example, the clinic's nurse practitioner offers primary health care services, and the clinic is located in the Community Health Centre, which offers additional services. Access to an infectious diseases physician is also available at the Uptown Clinic, where there is a focus on communicable disease prevention and management as a component of the medical model.

## Figures and Tables

**Table 1:** 

Population	Separated	Transfers from other programs	Induction phase	Eligible	Study sample
179 (100%)	9 (5%)	8 (4.5%)	28 (15.5%)	134 (75%)	84 (47%)
